# A Study of the Catalytic System H_3_PW_12_O_40_/Quaternary Phosphonium Salts for the Epoxidation of Fatty Acid Methyl Esters—The Effect of the Molar Ratio of Hydrogen Peroxide to the Double Bond

**DOI:** 10.3390/molecules30051109

**Published:** 2025-02-28

**Authors:** Marlena Musik, Ewa Janus, Robert Pełech

**Affiliations:** Department of Organic Chemical Technology and Polymer Materials, Faculty of Chemical Technology and Engineering, West Pomeranian University of Technology in Szczecin, Pułaskiego 10, 70-322 Szczecin, Poland; ewa.janus@zut.edu.pl (E.J.); robert.pelech@zut.edu.pl (R.P.)

**Keywords:** fatty acid methyl esters, epoxidation, quaternary phosphonium salts, hydrogen peroxide

## Abstract

In the present work, the epoxidation of fatty acid methyl esters (biodiesel or FAMEs) with an iodine number of 96.4 g/100 g and containing approximately 11% palmitic acid, 4% stearic acid, 51% oleic acid, 25% linoleic acid, and 5% linolenic acid was studied with an aqueous H_2_O_2_ solution and different quaternary phosphonium salts (QPSs) combined with the phosphotungstic heteropolyacid (HPA) H_3_PW_12_O_40_ in a biphasic system. The effect of the molar ratio of H_2_O_2_:C=C on the epoxidation of FAMEs was investigated. The effect of the molar ratio of H_2_O_2_:C=C on the epoxy number (EN) and iodine number (IN) was measured. Multiple regression analysis methods were used to determine the regression model describing the influence of the various independent variables. In the results obtained, it was found that the highest yields were obtained for [P6][Phosf]. The optimum conditions for the epoxidation process with the systems used were a time range of 30 ± 4 min and a H_2_O_2_/double bond molar ratio in the range of 1.8 ± 0.2. The formation of epoxidised fatty acid methyl esters (E-FAMEs) was confirmed by FT-IR, ^1^H NMR and ^13^C NMR analyses. In the FT-IR spectrum of the E-FAMEs, epoxy ring vibration signals were identified at 826 cm^−1^. In the ^1^H NMR spectrum, signals appeared in the range of 3.25–3.00 ppm, corresponding to epoxy ring formation in biodiesel, and in the range of 60–55 ppm in the ^13^C NMR spectrum.

## 1. Introduction

Epoxidised fatty esters and vegetable oils are widely used. Epoxidised soybean oil is a well-known example of a PVC plasticiser and stabiliser. Epoxidised fatty compounds are used in a wide variety of applications [1,2,3,4,5,6,7,8]. Other important uses include their use as intermediates in the production of various materials. Epoxides are powerful electrophiles and therefore readily form useful products: alkoxy alcohols, hydroxy thioethers, amino alcohols, vicinal diols, and hydroxy esters. In the manufacturing of polyurethane foams, polyols—formed when the rings of fatty epoxides are opened with water and alcohols—act as intermediates. Amino fatty alcohols have important anti-corrosion, anti-wear, and antioxidant properties. When epoxides react with acrylic acid, fatty epoxides called acrylates are formed. These monomers have reactive terminal double bonds and polymerise more readily than internal olefins to form polymers with a variety of physical properties [9].

Epoxidation is typically carried out via peracids in situ by reacting carboxylic acid (acetic or formic acid) with concentrated H_2_O_2_ [10,11]; another epoxidation catalyst is an acidic ion-exchange resin [12]. The catalysts used for epoxidation are very diverse [13,14,15,16,17].

One of the applications of phase-transfer catalysis (PTC) is epoxidation using hydrogen peroxide. H_2_O_2_ is the most common and environmentally friendly oxidant with a high content of active oxygen. It is a cheap, safe, and easy-to-use oxidant, and the only by-product is water [18]. Hydrogen peroxide is neutral to most organic substrates. The use of H_2_O_2_ in oxidation reactions requires the use of catalysts. The design of effective catalysts capable of heterolytic activation of the oxidant is an important issue in oxidation catalysis [19,20]. Phase-transfer catalysts play an important role in PTC systems. They transport the reactant from the primary phase to the phase containing the second reactant. A PT catalyst should be somewhat lipophilic so that it is soluble in both phases and can migrate between them [21].

Currently, several transition metal compounds have been investigated as homogeneous catalysts for the epoxidation of vegetable oils. Polyoxometalates (POMs) are a class of anionic metal oxygen clusters consisting of molybdenum(VI) [15], manganese [22], tungsten(VI) [23], and rhenium [24]. The properties of heteropolycatalysts from heteropolycomponents can be controlled by the appropriate selection of the polyanion and their components, such as heteroatoms, peripheral atoms, and counteractions. The study of POMs is of interest because of the variety of molecular structures and their applications in catalysis, medicine, and materials science. Polyoxometalates exhibit a wide variety of shapes, sizes and compositions, ranging from small (Mo_6_O_19_^2−^)^13^ to nanosized species (H_x_Mo_368_O_1032_(H_2_O)_240_(SO_4_)_48_^48−^) [25]. POMs can be converted to peroxopolyoxometalate (PPOM) compounds in solutions containing an excess of H_2_O_2_. PPOMs are active intermediates in epoxidation by H_2_O_2_ and can be considered as inorganic catalytic analogues of peracids [26]. The mechanism of the PTC reaction using H_2_O_2_ as an oxidant has been extensively described in publications [27,28,29]. PTC has many advantages in both laboratory and industrial applications, such as the elimination of organic solvents (anhydrous conditions are not required as water is used as one of the phases; this reduces the dependence of the reaction on organic solvents), the high reactivity of the active compounds (there is no need for aggressive conditions, resulting in a rapid reaction; also, high temperatures are not required, as the reaction usually takes place at low temperatures), the high yields and purity of the products, and the minimisation of industrial waste. The tunability of the molecular structures in the molecules of phase-transfer catalysts makes it possible to easily adjust the properties of the resulting catalysts, including their solubility, thus allowing controlled reactions, temperature responsiveness, etc. [28].

Research on polyoxometalate-based catalysts has developed very rapidly. Supported heteropolyacid catalysts are widely used in various organic transformations. One example is the work of Xie et al. [30]. The researchers aimed to develop catalysts that are desirable for biodiesel production. To this end, they developed a catalyst by incorporating ZIF-8 MOF into Fe_3_O_4_ nanoparticles and encapsulating a vanadium-substituted heteropolyacid (H_6_PV_3_MoW_8_O_40_). The resulting catalyst had good magnetic reactivity and could simultaneously catalyse the transesterification of soybean oil and the esterification of free fatty acid (FFA). The solid catalyst was recycled using an external magnet. The catalyst showed good recyclability. Another example is the research [31] to obtain a SnPW@ZIF-8 catalyst by modifying phosphotungstic acid (HPW) with tin metal and depositing it on organometallic ZIF-8. This catalyst was characterised by a large surface area with Brönsted–Lewis double acid sites, which increased the catalytic efficiency of oil transesterification and esterification of free fatty acids (FFAs) present in oils. This catalyst was also shown to be more resistant to water and FFA. This catalyst can be reused without compromising the efficiency of biodiesel production.

The mechanism of the epoxidation in the presence of a PT catalyst proposed by Ishii [32] concerns a system in which cetylpyridinium chloride acts as the PT catalyst, H_2_O_2_ as the oxidising agent, and H_3_PM_12_O_40_ as the oxidation catalyst. In this reaction, an active complex is formed when the heteropolyacid and PT catalyst interact in the aqueous phase. The pH of the reaction mixture is lowered as Cl^−^ anions from the pyridinium salt combine with H^+^ cations from H_3_PM_12_O_40_ to form the mineral acid HCl. Hydrogen peroxide is used to activate the complex of H_3_PM_12_O_40_ and the pyridinium salt. During this reaction, the oxygen atom from H_2_O_2_ attaches itself to the complex of H_3_PM_12_O_40_ and the pyridinium salt. The peroxocomplex migrates to the organic phase, where electrophilic addition occurs because the pyridinium salt cation is lipophilic. An oxygen from the catalyst complex binds to the olefin double bond. This produces an epoxide and a reduced version of the catalyst, which moves across the interface [21].

In the present work, selected quaternary phosphonium salts (QPS) in combination with phosphotungstic heteropolyacid H_3_PW_12_O_40_ and aqueous hydrogen peroxide solution were used to study the epoxidation of fatty acid methyl esters (biodiesel) in a biphasic system. Phosphonium polyoxometalate salts have advantageous properties, being characterised by very good thermal stability compared to other salts (ammonium or imidazole). The usefulness of such combinations in the epoxidation of fatty products has not been thoroughly investigated. The effect of the molar ratio of H_2_O_2_:C=C on the epoxidation of fatty acid methyl esters was studied. The following QPS were used in the study: trihexyltetradecylphosphonium tetrafluoroborate [P6][BF_4_], trihexyltetradecylphosphonium bis-(2,4,4-trimethyl-pentyl)-phosphinate [P6][Phosf], trihexyltetradecylphosphonium chloride [P6][Cl], tributyltetradecylphosphonium chloride [P4][Cl], and tetraoctylphosphonium bromide [P8][Br]. The effect of the H_2_O_2_:C=C molar ratio on the epoxy number and the iodine number was determined. Multiple regression analysis methods were used to determine the regression model describing the influence of the different independent variables. In the results obtained, it was found that the highest yields were obtained for [P6][Phosf]. The optimum conditions for the epoxidation process with the systems used were in the time range of 30 ± 4 min and with a H_2_O_2_/double bond molar ratio in the range of 1.8 ± 0.2.

## 2. Results

This study used an epoxidation method based on interfacial phase transfer catalysis, as presented in our previous publication [33]. The most favourable optimum temperature was found to be 50 °C, and this temperature was used in this study. The influence of the different types of QPS on the interfacial phase transfer catalysis epoxidation process was also investigated. The inactivity of the phosphonium salt trihexyltetradecylphosphonium bis(trifluoromethylsulfonyl)amide was found and is not considered in this publication. The epoxidation of FAMEs was carried out in a two-phase system: an oil layer and an aqueous layer. The heteropolyacid with the oxidant H_2_O_2_ was in the aqueous phase. When the complex entered the oil phase, it catalysed the cleavage of the double bond to form an epoxide ring.

The effect of the H_2_O_2_/unsaturated bond ratio on the epoxidation of FAMEs at 50 °C and reaction times from 5 to 30 min using different QPS was determined. The constant parameters were set as follows: the amount of substrate (FAMEs) was 17 mmol (0.0184 mol C=C), 1100 rpm, QPS:HPA = 3:1 (mmol/mmol), where HPA was phosphotungstic heteropolyacid.

**Figure 1:**
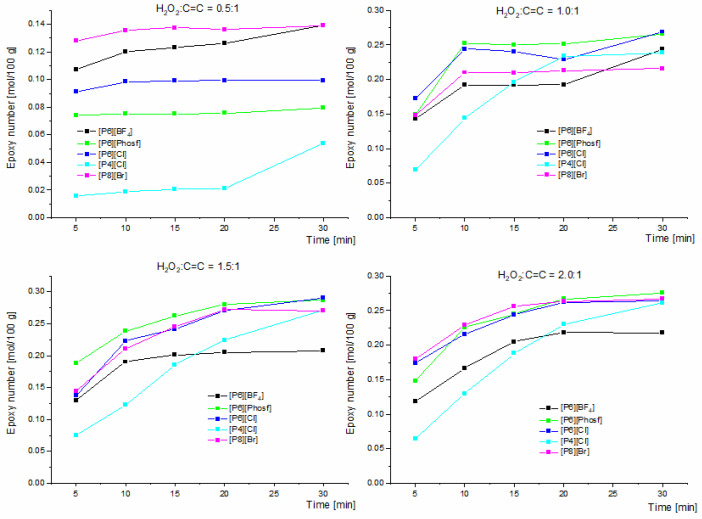
Effect of different H_2_O_2_:C=C molar ratios on the epoxide number as a function of time using different quaternary phosphonium salts in the epoxidation reaction of biodiesel by phase-transfer catalysis. Reaction conditions: the substrate (fatty acid methyl esters) amount was 17 mmol (0.0184 mol C=C). QPS:HPA = 3:1 (mmol/mmol).

**Figure 2:**
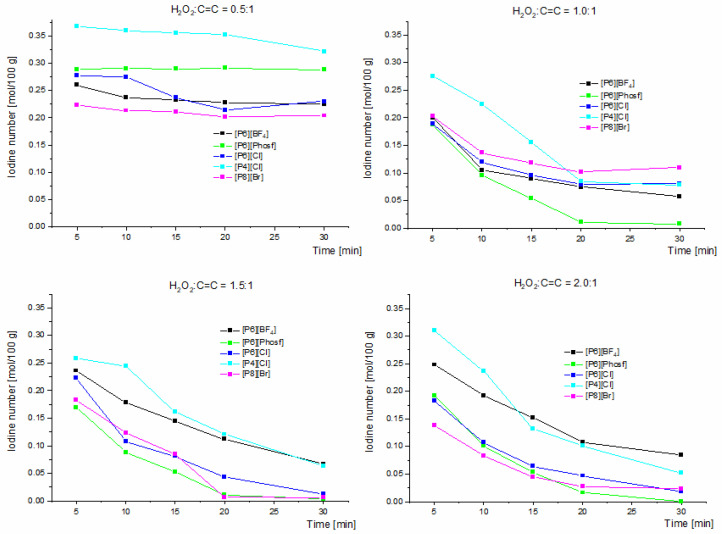
Effect of different H_2_O_2_:C=C molar ratios on the iodine number as a function of time using different quaternary phosphonium salts in the epoxidation reaction of biodiesel via phase-transfer catalysis for the substrate (FAMEs) amount 17 mmol (0.0184 mol C=C) and QPS:HPA = 3:1 (mmol/mmol).

The results presented in Figure 1 and Figure 2 illustrate the effect of different molar ratios of hydrogen peroxide on the unsaturated bond. The results are presented within a statistical error of ±2% and include data obtained for all the proposed salts at different H_2_O_2_:C=C ratios.

The results indicate that increasing the concentration of H_2_O_2_ to the unsaturated bond results in increasing epoxide numbers. Considering Figure 1 for the lowest molar ratio of 0.5:1, the highest epoxy number was obtained after 30 min EN = 0.139 mol/100 g using the following phosphonium salts: [P8][Br] and [P6][BF_4_]. The lowest values of epoxide number EN = 0.054 mol/100 g were obtained for the salt [P4][Cl]. By increasing the molar ratio to 1.0:1, very similar epoxide number values were obtained for [P6][Phosf] and [P6][Cl] salts, being 0.266 and 0.269 mol/100 g, respectively. Increasing the molar ratio resulted in greater activation of the [P4][Cl] salt, where the epoxide number after 30 min of running the process was 0.239 mol/100 g. A further increase in the H_2_O_2_:C=C molar ratio to 1.5:1 resulted in very similar epoxide numbers for the three phosphonium salts [P6][Phosf]—EN = 0.287 mol/100 g, [P6][Cl]—EN = 0.291 mol/100 g, and [P8][Br]—0.270 mol/100 g. At a molar ratio of H_2_O_2_:C=C of 2.0:1, the highest epoxy numbers after 30 min were obtained with [P6][Phosf] EN = 0.276 mol/100 g and [P6][Cl] EN = 0.265 mol/100 g, and the epoxy number was EN= 0.267 mol/100 g when using [P8][Br] salt. For this molar ratio, the highest value of EN= 0.218 mol/100 g was obtained for the [P6][BF_4_] salt after 30 min. Regarding interfacial transfer catalysed epoxidation reactions using the [P4][Cl] salt, very similar trends were obtained at H_2_O_2_:C=C molar ratios of 1.5:1 and 2.0:1. For this salt, an increase in reaction time may be crucial.

These relationships are supported by changes in IN as a function of time (Figure 2). At a molar ratio of H_2_O_2_:C=C of 0.5:1 using phosphonium salt [P4][Cl], the iodine number decreased from a value of 0.370 mol/100 g to 0.322 mol/100 g. For the phosphonium salt [P6][Phosf], the iodine number was constant throughout the process at IN = 0.290 mol/100 g. The lowest values of IN were obtained with the phosphonium salt [P8][Br] at 5 min after the start of epoxidation; it reached 0.220 mol/100 g, while after 30 min, it was 0.200 mol/100 g. Larger changes were observed with the salt [P6][Cl]; the IN decreased from 0.280 mol/100 g to 0.23 mol/100 g. Also, for H_2_O_2_:C=C molar ratios of 1.0:1, 1.5:1 and 2.0:1, a decrease in the iodine value to 0 was observed with the use of [P6][Phosf], which could indicate that the double bonds were filled with oxirane oxygen or the formation of undesirable by-products such as diols (this was confirmed by the appearance of a band around 3500 cm^−1^ in the FT-IR spectrum). This effect was caused by a higher proportion of subsequent reactions towards glycol in this catalytic system.

In the data obtained, the optimum conditions for each ionic liquid were determined. Multiple regression analysis methods were used to determine the regression model describing the influence of each independent variable. Statistical analyses were performed on the real data. The following regression equation in the form of a second-degree polynomial was used for the systems studied:

(1) $$Y=a_{0}+\sum_{i=1}^{2}a_{i1}\cdot X_{i}+\sum_{i=1}^{2}a_{i2}\cdot X_{i}^{2}+a_{3}\cdot X_{1}\cdot X_{2}$$

where X_i_—molar ratio H_2_O_2_:C=C, epoxidation reaction time in min, and Y—yield.

**Figure 3:**
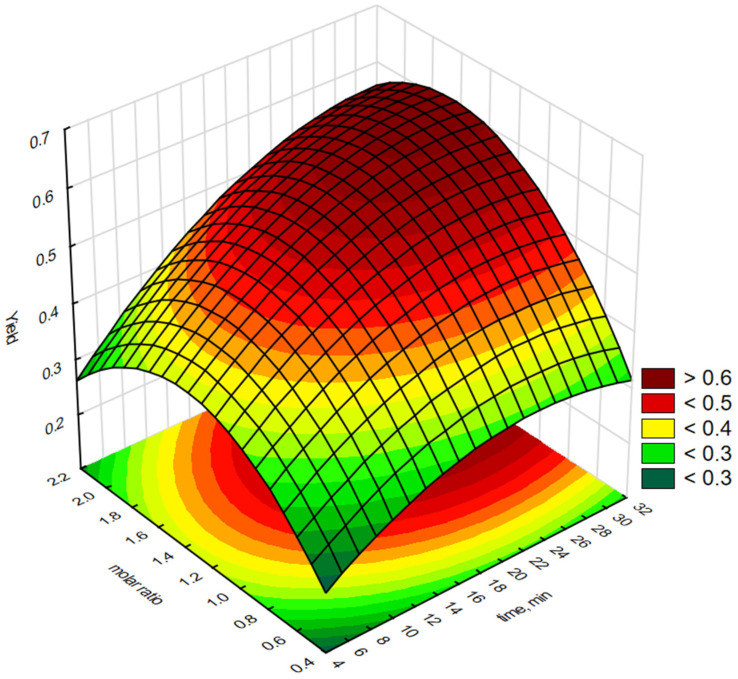
Response surface plot showing the reciprocal effect of H_2_O_2_:C=C molar concentration and epoxidation reaction time on epoxidation efficiency using interfacial transfer catalysis in the presence of a phosphonium salt [P6][BF_4_].

**Figure 4:**
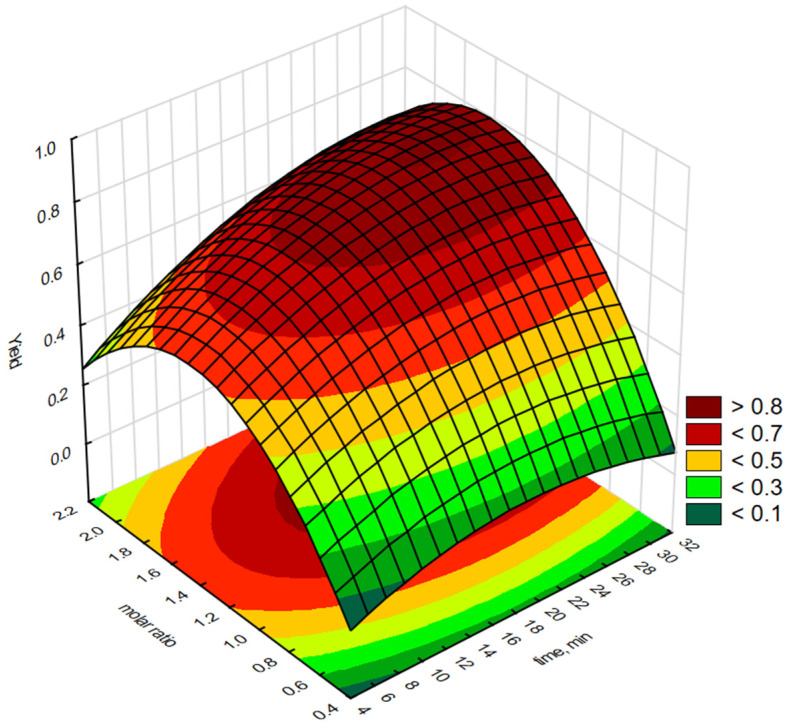
Response surface plot showing the reciprocal effect of H_2_O_2_:C=C molar concentration and epoxidation reaction time on epoxidation efficiency using interfacial transfer catalysis in the presence of a phosphonium salt [P6][Phosf].

**Figure 5:**
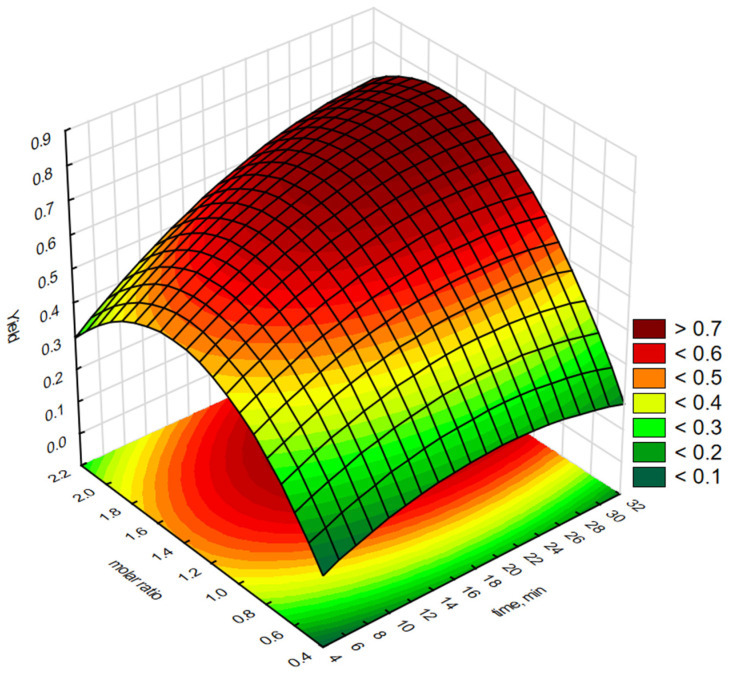
Response surface plot showing the reciprocal effect of H_2_O_2_:C=C molar concentration and epoxidation reaction time on epoxidation efficiency using interfacial transfer catalysis in the presence of a phosphonium salt [P6][Cl].

**Figure 6:**
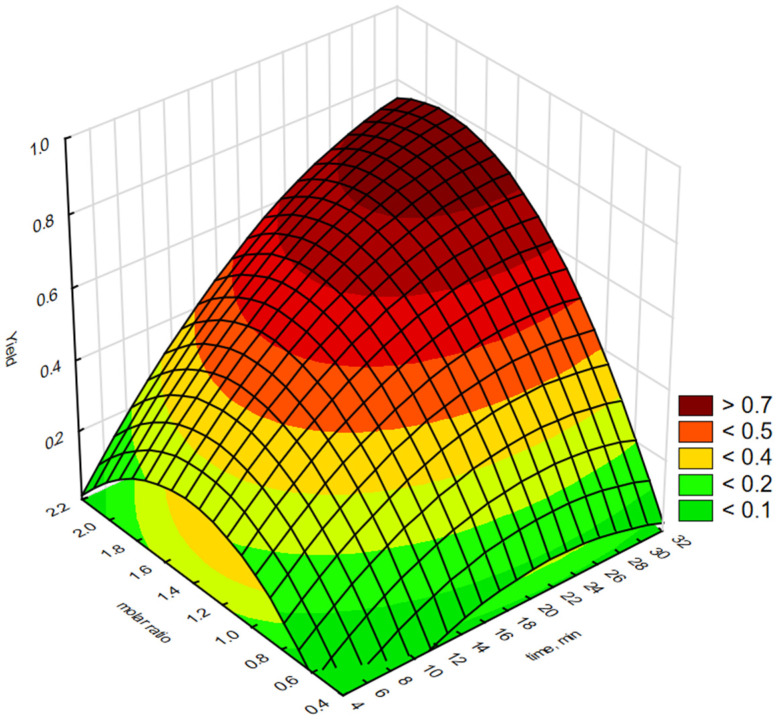
Response surface plot showing the reciprocal effect of H_2_O_2_:C=C molar concentration and epoxidation reaction time on epoxidation efficiency using interfacial transfer catalysis in the presence of a phosphonium salt [P4][Cl].

**Figure 7:**
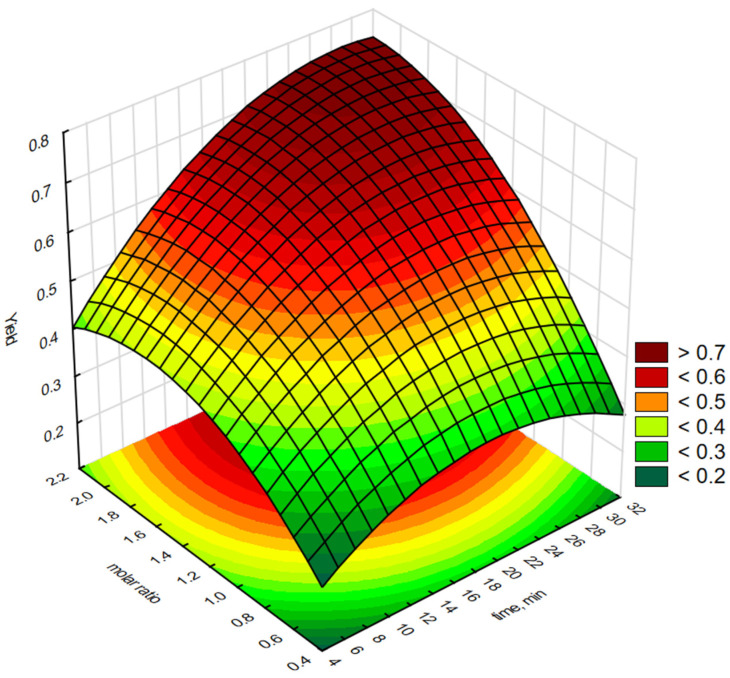
Response surface plot showing the reciprocal effect of H_2_O_2_:C=C molar concentration and epoxidation reaction time on epoxidation efficiency using interfacial transfer catalysis in the presence of a phosphonium salt [P8][Br].

Statistica software 13.3 was used to generate graphical representations of a regression Equation (1) and 3-D response surface. Figure 3, Figure 4, Figure 5, Figure 6 and Figure 7 illustrate the relationship between the independent and dependent variables for different fixed parameters. These plots offer a clear visualisation of how the responses correlate with the test variables.

Figure 3 shows the influence of the time (X_1_) and the molar ratio of H_2_O_2_:C=C (X_2_) on the yield of epoxidation fatty acid methyl esters using [P6][BF_4_]. As shown in Figure 3, the yield of epoxidised biodiesel increased as the molar ratio of H_2_O_2_:C=C increased. The yield reached a maximum at a H_2_O_2_:C=C molar ratio between 1.6:1 and 1.8:1 and then decreased in the range from 1.8:1 to 2.2:1. Further increases in the H_2_O_2_:C=C molar ratio result in over-oxidisation or repolymerisation of a small fraction of the products to small molecules or polymers [34]. Maximum epoxidation efficiency (>60%) was observed when the H_2_O_2_:C=C molar ratio was 1.6–1.8 after 30 min.

The effect of reaction time (X_1_) and H_2_O_2_:C=C molar ratio (X_2_) on the epoxidation yield of biodiesel using a QPS in the form of [P6][Phosf] is shown in Figure 4. The epoxidation yield of FAMEs increased with an increasing reaction time. However, increasing the molar ratio of H_2_O_2_:C=C above 1.8:1 resulted in a slight decrease in the epoxidation yield, as a higher molar ratio of H_2_O_2_:C=C could lead to rapid dissociation of H_2_O_2_ or decomposition of the resulting epoxides [35]. The maximum epoxidation yield with this salt was obtained at H_2_O_2_:C=C molar ratios of 1.2:1 to 1.8:1 and reaction times of 26 min.

Figure 5 shows the effect of the reaction time (X_1_) and H_2_O_2_:C=C molar ratio (X_2_) on the epoxidation yield of biodiesel using the quaternary phosphonium salt as [P6][Cl]. The yield of biodiesel increased with increasing reaction time. The peak was reached at a reaction time of 30 min. The maximum epoxidation yield was obtained at a moderate H_2_O_2_:C=C molar ratio (1.2–1.7) and optimum reaction time (30 min).

Figure 6 shows the effect of the reaction time (X_1_) and H_2_O_2_:C=C molar ratio (X_2_) on the epoxidation yield of biodiesel using a quaternary phosphonium salt in the form of [P4][Cl]. The efficiency was increased by increasing the reaction time from 24 to 32 min. Reaction values peaked and were most efficient near H_2_O_2_:C=C molar ratios of 1.4:1 to 2.2:1 and reaction times above 24 min.

Figure 7 shows the effect of the reaction time (X_1_) and H_2_O_2_:C=C molar ratio (X_2_) on the epoxidation yield of biodiesel using QPS in the form of [P8][Br]. Increasing the reaction time resulted in a remarkable increase in the yield. Both the reaction time and the H_2_O_2_:C=C molar ratio had a positive effect on the yield. In general, the maximum epoxidation yield was obtained at a reaction time of about 29 min and a H_2_O_2_:C=C molar ratio of 2.2:1.

A second-order polynomial regression equation was solved to determine the optimal critical values for the epoxidation process using interfacial transfer catalysis under different quaternary phosphonium salts. The optimum conditions for epoxidation using different ionic liquids are shown in Table 1.

The regression coefficients, together with the correlation coefficient of the area to the experimental points, are shown in Table 2 below.

**Figure 8:**
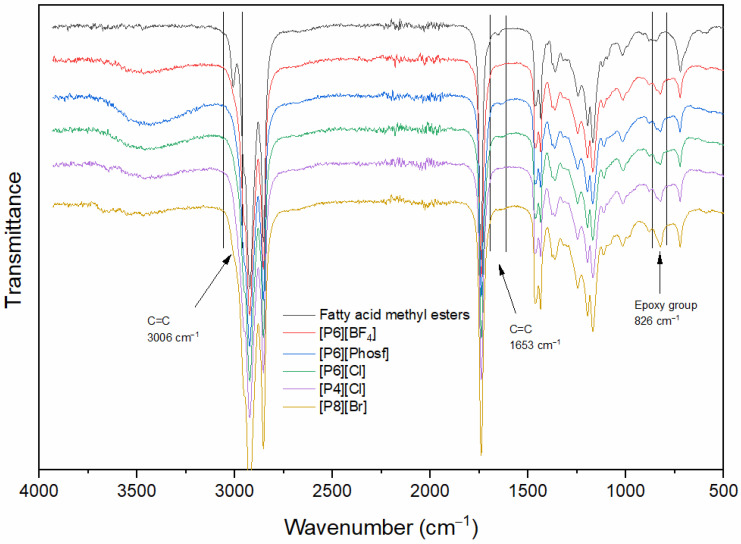
FTIR analysis of FAMEs and the epoxidation products using five QPS after 30 min of reaction. Reaction conditions: 17 mmol of FAMEs (0.0184 mol of C=C); H_2_O_2_:Biodiesel = 2.0:1; H_2_O_2_:C=C:HPA = 2.0:1:0.0042; [QPS]:HPA = 3:1.

FT-IR spectra were obtained for both the FAMEs and the corresponding epoxides, in agreement with those reported by various authors [36,37,38]. The most representative vibration signals of the fatty acid methyl esters corresponded to the ester carbonyl (1739 cm^−1^) (C=O) and double-bound signals at 3006 cm^−1^ (=C-H), 1653 cm^−1^ (C=C) and 722 cm^−1^ (HC=CH). The other vibration bands corresponded to ester carbonyl (1739 and 1169 cm^−1^), methyl (2923, 1435 and 1361 cm^−1^), and methylene (2853 cm^−1^) groups. In the E-FAME spectrum, the vibration signals of the epoxy ring were identified at 826 cm^−1^ (C-O-C). The signal of C=C bending at 3006 cm^−1^ was noted in the spectrum of fatty acid methyl esters, which disappeared in the spectrum of E-FAMEs. The results showed the removal of carbon-carbon double bonds and the formation of epoxy groups following the epoxidation reaction. In addition, the appearance of a broad band at 3500 cm^−1^, characteristic of O-H stretching vibrations, was observed, indicating the formation of alcohols in the product, which would imply the opening of the epoxy ring. Samples were taken for FTIR analysis after 30 minutes of the epoxidation reaction (Figure 8).

**Figure 9:**
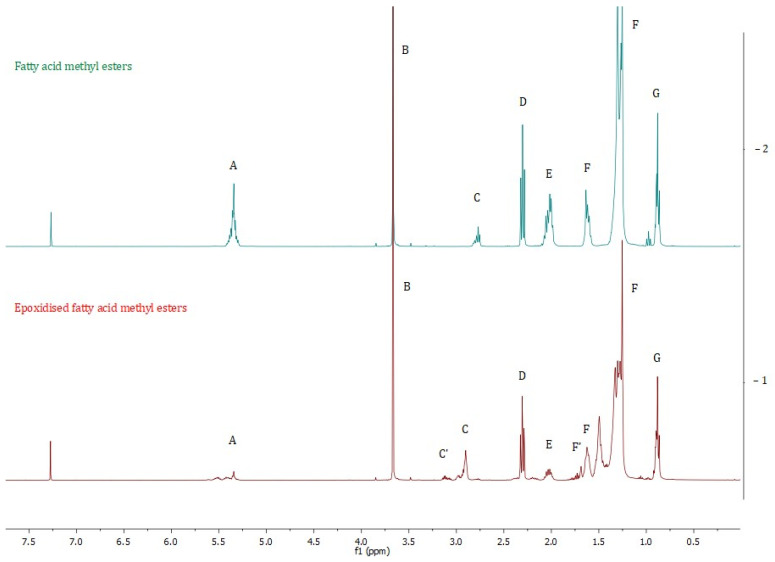
^1^H NMR spectra of fatty acid methyl esters (−2) and epoxidised fatty acid methyl esters (−1). An explanation of the signals is provided in Table 3.

**Figure 10:**
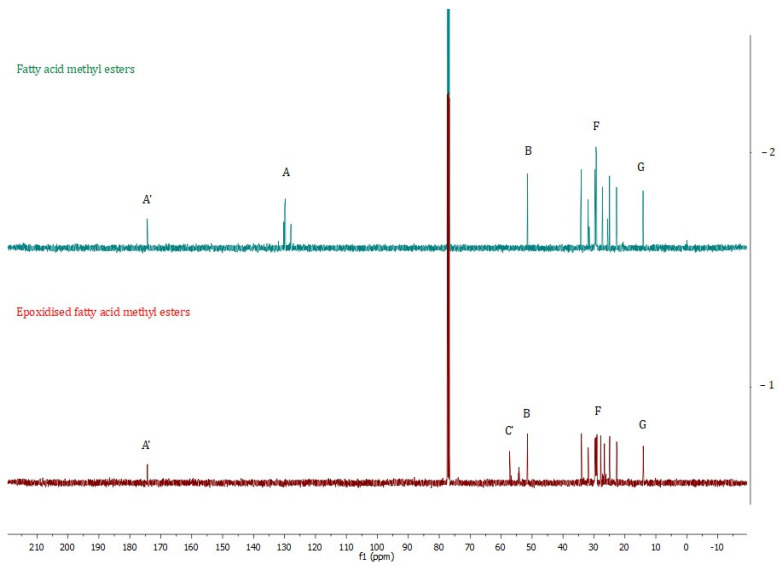
^13^C NMR spectra of fatty acid methyl esters (−2) and epoxidised fatty acid methyl esters (−1). An explanation of the signals is provided in Table 3.

In Table 3 and Figure 9 and Figure 10, different groups of signals indicative of epoxide groups were found in epoxidised fatty acid methyl esters [39,40].

## 3. Materials and Methods

### 3.1. Materials

FAMEs from rapeseed oil with iodine number 96.4 g/100 g were used in the study. The composition of the FAMEs, determined using gas chromatography–mass spectrometry analysis, was (% *w*/*w*) palmitic = 11.1, stearic = 3.5, oleic = 51.2, linoleic = 25.1, and linolenic = 4.8. Hydrogen peroxide (30%) (Stanlab, Łódź, Poland), phosphotungstic acid hydrate H_3_PW_12_O_40_ aq < 0.02% (Lach-ner, Neratovice, Czech Republic), tetradecyltrihexylphosphonium bis(2,4,4-trimethylpentyl)phosphinate (Fluka, Steinheim, Germany), tributyltetradecylphosphonium chloride (≥95.0% Fluka, Steinheim, Germany), trihexyltetradecylphosphonium tetrafluoroborate (Fluka, Steinheim, Germany), tetradecyltrihexylphosphonium chloride (≥95% Fluka, Steinheim, Germany), tetraoctylphosphonium bromide (97% Sigma Aldrich, Steinheim, Germany), TEA bromide C_8_H_20_NBr (MERCK, Darmstadt, Germany), crystal violet indicator, 0.1 N PCA HClO_4_ (Sigma Aldrich, Steinheim, Germany), 0.1 N sodium thiosulfate Na_2_S_2_O_3_ (Eurochem, Tarnów, Poland), dichloromethane CH_2_Cl_2_ (POCh, Gliwice, Poland), chloroform CHCl_3_ (Stanlab, Lublin, Poland), glacial acetic acid (POCh, Gliwice, Poland), Hanus solution (IBr) (ROTH, Karlsruhe, Germany), starch (Fluka, Steinheim, Germany), and potassium iodide (Fluka, Seelze, Germany).

### 3.2. Epoxidation of Biodiesel

Phosphotungstic acid H_3_PW_12_O_40_ and hydrogen peroxide were placed in a 25 cm^3^ round bottom three-necked flask equipped with thermometer, mechanical stirrer, and reflux condenser. The flask containing the solution was immersed in an water bath with temperature control. The mixture was stirred at 1100 rpm for 15 min at the reaction temperature of 50 °C. After this time, the appropriate quaternary phosphonium salt was added. Stirring was resumed for 10 min. Finally, the FAMEs were added. The process was carried out with vigorous stirring at 1100 rpm. The reaction mixture was sampled at the following time intervals after—5, 10, 15, 20, and 30 min. The molar ratio of H_2_O_2_:C=C was varied from 0.5:1, 1.0:1, and 1.5:1 to 2.0:1. The mixture taken from the solution consisted of E-FAMEs together with H_2_O_2_ and H_2_O after the reaction was centrifuged for 10 min at 7000 rpm to separate the organic phase from the aqueous phase and then analysed. IN and EN were determined to monitor the progress of epoxidation reaction and oxirane cleavage [33].

### 3.3. Methods

#### Identification of the Biodiesel by the Gas Chromatography Method

Qualitative and quantitative analyses of biodiesel were carried out using chromatographic methods. The FAMEs were carried out using a Thermo Electron GC FOCUS chromatograph (Thermo Company, Waltham, MA, USA). The parameters of the analyses were the same as in our previous publication [33]. In addition, qualitative chemical analyses were performed using a GC–MS ThermoQuest series instrument with a VOYAGER mass detector (Agilent, Santa Clara, CA, USA).

### 3.4. Epoxide Number

The epoxide number (EN) before and after epoxidation was determined according to EN ISO 3001 [41]. Samples of methyl esters of fatty acids were dissolved in chloroform. Then, 10 mL of tetraethylammonium bromide solution and a few drops of indicator were added. The mixture was then titrated with perchloric acid to a blue-green colour. A blank test was carried out in the same way but without fatty acid methyl esters. The epoxy number for raw materials and epoxidised biodiesel was determined:

$$EN=\frac{(A-C)\cdot 0.1\cdot 100}{W\cdot 1000}$$

with regard to the following:

EN is the epoxy number of the sample (mol/100 g)
A is to the volume of HClO_4_ required for titration of sample (mL)
C is to the volume of HClO_4_ required for titration of blank solution (mL).

### 3.5. Iodine Number

The Hanus method was used to determine iodine number (IN) [42]. The degree of unsaturation of fatty acid methyl esters can be measured by the iodine number, which is the number of grams of iodine consumed in a reaction with 100 g of fatty acid methyl esters. The samples were dissolved in 10 mL of chloroform and 25 mL of Hanus solution was added and left in dark for 30 min. Then, 20 mL of KI solution and 250 mL of distilled water were added and titrated with 0.1N sodium thiosulfate. When the solution turned yellow, 1 mL of starch solution was added and the titration continued until the blue colour disappeared. The iodine number was then calculated using Hanus equation:

$$IN=\frac{\left[\left(B-V\right)\cdot N\cdot 12.69\right]}{W}$$

where the following apply:

IN is iodine number of the sample (mol/100 g);
B is the volume of Na_2_S_2_O_3_ required for titration of blank solution (mL);
V is the volume of Na_2_S_2_O_3_ required for titration of sample (mL);
N is normality of Na_2_S_2_O_3_ solution (0.1 N);
W is mass of sample used (g) for titration.

### 3.6. ^1^H NMR and ^13^C NMR Spectroscopy

^1^H NMR spectra were obtained using a Bruker DPX-400 Avance III HD spectrometer (Bruker, Billerica, MA, USA) operating at 400.13 MHz (^1^H) and 100.62 MHz (^13^C). Sam-ples were dissolved in CDCl_3_. The deuterated chloroform chemical shift peak at 7.26 ppm was used as an internal reference.

### 3.7. Fourier Transformed Infrared Spectroscopy (FTIR)

FTIR spectra of the final products were recorded on a Thermo Fisher Scientific Nicolet 380 FT-IR Spectrometer (Waltham, MA, USA). The FTIR spectra were obtained by placing the samples directly on an Attenuated Total Reflectance (ATR) element (diamond crystal) in the 4000–400 cm^−1^ frequency range (ATR) with 128 scans and a resolution of 1 cm^−1^.

## 4. Conclusions

The epoxidation of FAMEs from rapeseed oil was carried out using an aqueous H_2_O_2_ solution and different QPS with the H_3_PW_12_O_40_. A regression model describing the influence of each independent variables was determined. Multiple regression analysis was used. Critical optimal values were determined for the quaternary phosphonium salts used. The regression coefficients were presented together with the correlation coefficient of the area with the experimental points. In the data obtained, the optimum conditions for each ionic liquid were determined. It was found that the highest performance was obtained using the quaternary phosphonium salt [P6][Phosf]. The optimum conditions for the epoxidation process with the systems used are in the time range of 30 ± 4 min and with a molar ratio of H_2_O_2_ to oil in the range of 1.8 ± 0.2. In the FT-IR spectrum of E-FAMEs, the vibration signals of the epoxy ring were identified at 826 cm^−1^. In the ^1^H NMR spectrum of the epoxidised products, signals appeared in the range of 3.25–3.00 ppm, corresponding to epoxide ring formation in biodiesel and 60–55 ppm in the ^13^C NMR spectrum.

## Figures and Tables

**Table 1:** Critical optimum values for individual liquids.

QPS					
Parameter	[P6][BF_4_]	[P6][Phosf]	[P6][Cl]	[P4][Cl]	[P8][Br]
Reaction time t, min	30	27	30	35	29
H_2_O_2_:C=C molar ratio	1.6	1.6	1.7	1.9	2.2
Epoxidation yield, Y	0.61	0.81	0.78	0.79	0.75

**Table 2:** Regression coefficients with correlation coefficient of area to experimental points.

Coefficient	[P6][BF_4_]	[P6][Phosf]	[P6][Cl]	[P4][Cl]	[P8][Br]
a_0_	−0.026549	−0.501043	−0.290242	−0.586270	0.004935
a_11_	0.015580	0.019218	0.015697	0.025894	0.019021
a_12_	−0.000368	−0.000563	−0.000447	−0.000682	−0.000551
a_21_	0.499677	1.306881	0.991561	0.972148	0.437105
a_22_	−0.186080	−0.464831	−0.353320	−0.357916	−0.140858
a_3_	0.003776	0.006953	0.006572	0.011343	0.005885
R^2^	0.85	0.94	0.90	0.91	0.93
R^2^ adjusted	0.80	0.91	0.86	0.88	0.91
Mean squared error	0.0024	0.0037	0.0048	0.0074	0.0018

**Table 3:** ^1^H and ^13^C NMR chemical shifts observed from fatty acid methyl esters and epoxidised fatty acid methyl esters.

**Fatty Acid Methyl Esters**			
**Signal**	**Moietie**	**Chemical Shifts ppm**	
		**^1^H NMR**	**^13^** **C NMR**
A′	C=O	–	174.36
A	–C**H**=C**H**– (–C**H**=CH–CH=C**H**–)	5.35	130.24–127.93
B	Methyl ester –CH_3_	3.67	51.46
C	–CH_2_– between two non-conjugated double	2.78	-
D	–CH_2_– adjacent to the carbonyl group	2.30	-
E	–CH_2_– adjacent to the double bonds	2.03	-
F	The aliphatic –CH_2_–s	1.62	34.12–22.60
		1.29	
G	End of chain aliphatic –CH_3_	0.88	14.13
**Epoxidised fatty acid methyl esters**			
**Signal**	**Moietie**	**Chemical shifts ppm**	
		**^1^H NMR**	**^13^** **C NMR**
A	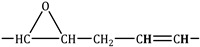 olefinic group to epoxy group	5.50–5.30	-
B	Methyl ester –CH_3_	3.60	51.50
C	–CH_2_– between two non-conjugated double	2.83	-
C’	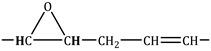 epoxy group to olefinic group	3.25–3.00	60.00–55.00
D	–CH_2_– adjacent to the carbonyl group	2.24	-
E	–CH_2_– adjacent to the double bonds	1.95	-
F	The aliphatic –CH_2_–s	1.54	34.30–22.59
		1.27	
F′	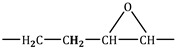	1.43	-
G	End of chain aliphatic –CH_3_	0.82	14.14

## Data Availability

The original contributions presented in the study are included in the article; further inquiries can be directed to the corresponding authors.
